# Calcined Oyster Shell Powder as a Natural Preservative for Maintaining Quality of White Shrimp (*Litopenaeus vannamei*)

**DOI:** 10.3390/biology11020334

**Published:** 2022-02-20

**Authors:** Wen-Chien Lu, Chien-Shan Chiu, Chang-Wei Hsieh, Yung-Jia Chan, Zeng-Chin Liang, Chiun-C. Roger Wang, Amanda Tresiliana Mulio, Dung Huynh Thi Le, Po-Hsien Li

**Affiliations:** 1 Department of Food and Beverage Management, Chung-Jen Junior College of Nursing, Health Sciences and Management, 217, Hung-Mao-Pi, Chia-Yi City 60077, Taiwan; m104046@cjc.edu.tw; 2 Department of Dermatology, Taichung Veterans General Hospital, 1650 Section 4 Taiwan Boulevard, Xitun District, Taichung 40705, Taiwan; chienshan@vghtc.gov.tw; 3 Department of Food Science and Biotechnology, National Chung Hsing University, 145 Xingda Road, South District, Taichung 40227, Taiwan; welson@nchu.edu.tw; 4 College of Biotechnology and Bioresources, Da-Yeh University, 168, University Road, Dacun, Changhua 51591, Taiwan; chanyungjia@gmail.com; 5 Department of Medicinal Botanical and Health Applications, Da-Yeh University, 168, University Road, Dacun, Changhua 51591, Taiwan; zcliang@mail.dyu.edu.tw; 6 Department of Food and Nutrition, Providence University, 200, Section 7, Taiwan Boulevard, Shalu District, Taichung City 43301, Taiwan; jcwang@pu.edu.tw (C.-C.R.W.); tresiliana@gmail.com (A.T.M.); 7 Faculty of Food Science and Technology, Ho-Chi-Minh City University of Food Industry, 140, Le Trong Tan Street, Tay Thanh Ward, Tan Phu District, Ho-Chi-Minh City 700000, Vietnam; dunghtl@hufi.edu.vn

**Keywords:** white shrimp, natural preservatives, calcined oyster shell powder, total volatile basic nitrogen, shelf-life

## Abstract

**Simple Summary:**

The Food and Agriculture Organization of the United Nations (FAO) indicated that the average global production of oyster shell waste for the year 2019 was 3.08 million tons. Many serious problems include the emission of displeasing odors and pollution of the seaside, which are harmful to the environment. Nonetheless, a solution for this issue would be to reuse the waste and produce a product that has economic benefits and solves the environmental problems. Using calcined oyster shells as a natural preservative might solve the problem of oyster shell waste. In this study, we used calcined oyster shell powder (COSP) as a natural preservative for improving shrimp shelf-life during 12 days under refrigerated conditions. As compared with the control, COSP treatment effectively retarded pH change, reduced the formation of total volatile basic nitrogen, and inhibited bacterial growth during refrigerated storage. The development of preservatives for aquatic products is expected to delay the growth of and spoilage by microorganisms in the refrigerated state, thus providing more barrier protection for aquatic food safety.

**Abstract:**

Oyster shell waste has led to many problems, including displeasing odors, pollution of the seaside, and harm to the environment. Using calcined oyster shells as a natural preservative might solve the problem of oyster shell waste. We studied the use of calcined oyster shell powder (COSP) as a natural preservative for improving shrimp shelf-life over 12 days under refrigerated conditions. As compared with the control, COSP treatment effectively retarded pH change, reduced the formation of total volatile basic nitrogen, and inhibited bacterial growth during refrigerated storage. In addition, shrimp muscle lipid oxidation measured by peroxide value (PV) and thiobarbituric acid (TBA) was decreased during storage. The quality was preserved up to 12 days with 2.0–4.0% COSP treatment as compared with only 6 days for un-treated shrimp. The development of preservatives for aquatic products is expected to delay growth of and spoilage by microorganisms in the refrigerated state, thus providing more barrier protection for aquatic food safety.

## 1. Introduction

Seafoods, such as fish and shellfish, are highly perishable and have a limited shelf-life after capture because of their biological composition. To avoid microbiological spoilage and enzymatic reactions, a low temperature is one of the most vital criteria, being able to reduce enzyme and microorganism activity [1]. The use of temperature regulation to preserve aquatic products is significant worldwide for both the local and export markets. Refrigerated storage is a dependable coolant that has been put to good use for centuries for cooling seafood and aquatic products both on- and offshore. Even though refrigerated storage has a bacteriostatic effect on food spoilage, it still does not entirely inhibit microbial reactions in the seafood matrix. Preservatives, such as antioxidant additives, oxygen or ion chelating agents, and antibacterial compounds, may be applied to maintain shelf-life and quality of seafoods. Therefore, the use of natural preservatives to extend the shelf-life of seafood and inhibit foodborne pathogens has received much attention [2]. Attention to natural preservatives as aquatic-product preservative agents is mostly determined by consumer’s demands for more natural preservatives, no additives, and protection of the environment [3].

Recently, there has been an interesting development of reuse materials with antimicrobial properties which help to improve food safety and shelf-life. Calcined oyster shell powder (COSP) might have antimicrobial activity potential by retarding microbial growth [4]. A previous study revealed that reused oyster shell powder (0.05, 0.1, and 0.2%) extended the shelf-life of tofu by up to 2 days during storage and enhanced its sensory value and quality; also, the microbial number was greatly reduced, which preserved tofu quality and freshness [5]. The addition of 0.05% reused oyster powder significantly enhanced the quality of kimchi during storage time; it retarded aerobic bacteria proliferation and preserved lactic acid bacteria proliferation, thus resulting in longer shelf-life while maintaining the sensory quality and crispiness of kimchi [6].

The Food and Agriculture Organization of the United Nations (FAO) indicated that the average global production of oyster shell waste for the year 2019 was 3.08 million tons, and the average output of oyster shells in Taiwan is >160,000 tons annually [7,8]. Many serious problems include the emission of displeasing odors and pollution of the seaside, which are harmful to the environment. Nonetheless, a solution for this issue would be to reuse the waste and produce a product that is economically profitable and solves the environmental problems. Lack of effective management on fishing boats, such as the working time, number of nets, and primary sorting and processing on the deck, leads to insufficient temperature control in the fishing boat cabin. This situation may increase the risk of aquatic spoilage bacteria and pathogenic bacteria attachment, breeding, and cross-contamination [9,10]. Discussion of the issue of aquatic food safety has increased in recent years. Hence, the use of natural food preservatives instead of synthetic preservatives. The aim of this study was to evaluate the effect of COSP on the physicochemical properties of white shrimp (*Litopenaeus vannamei*) under refrigerated storage conditions.

## 2. Materials and Methods

### 2.1. Materials

White shrimp (*L. vannamei*) with an average weight of 12.45 ± 1.60 g and length of 15.82 ± 0.68 cm were purchased from a local market (Wuqi Harbor, Taichung, Taiwan). The shrimp were kept in an insulated polyethylene box with a ratio of shrimp to water of 1:2 (*w*/*w*) and transported to the laboratory within 2 h.

### 2.2. Preparation of COSP

The oyster shell waste used in this study was sourced from Wuqi Harbor. The oyster shells were brushed and soaked in water for a week or more to remove salt and foreign substances. Washed oyster shells were naturally dried and then crushed. Crushed oyster shells were calcined in an electric furnace at 900 °C for 3 h. COSP was slowly cooled to room temperature in the electric furnace, then pulverized to pass through a 150 µm sieve. All chemical reagents used for extraction and analysis were of analytic grade.

### 2.3. Dipping Solutions and Refrigerated Storage Conditions for Shrimp

**Figure 1:**
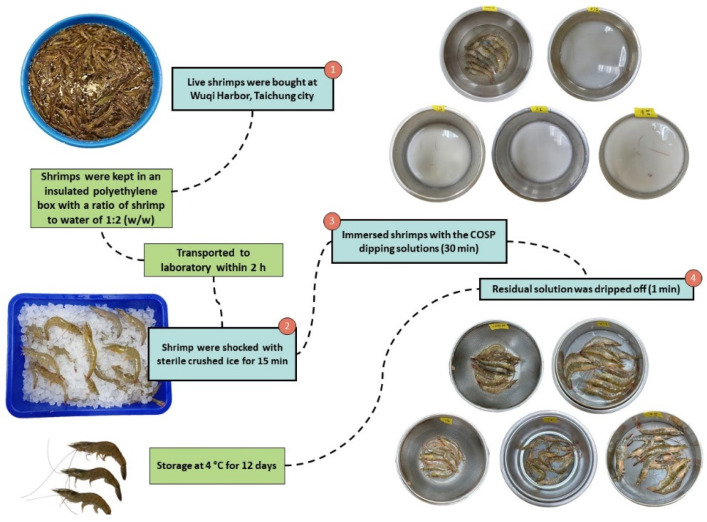
Shrimp samples prepared using different concentrations of COSP dipping solutions.

The COSP proportion in dipping solutions was 0.5%, 1%, 2%, and 4% in sterile water, respectively. The shrimp were shocked with sterile crushed ice for 15 min and fully immersed in the four dipping solutions for 30 min at 4 °C. Residual solution was dripped off for 1 min and the shrimp were kept at 4 °C until excess water was drained. Then shrimp were placed on plastic-coated wire racks inside plastic containers at 4 °C for 12 days (Figure 1).

### 2.4. COSP Characterization

The ash (AOAC 923.03), moisture (925.10), carbohydrate (AOAC997.08), and protein (AOAC 979.09) contents in COSP samples were analyzed according to standard methods described by the AOAC (2000) [11].

### 2.5. Biochemistry Quality Evaluation

**Figure 2:**
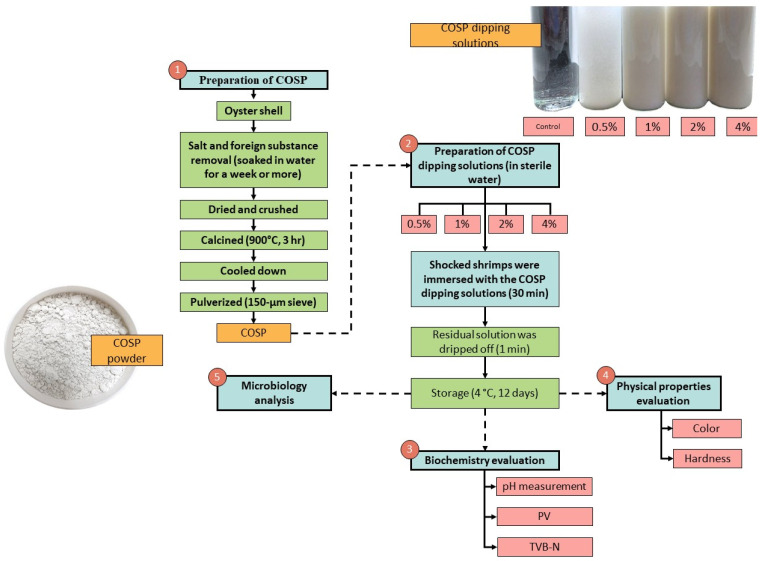
The experimental design of white shrimp treated with different concentrations of COSP dipping solutions.

The evaluation of biochemistry quality was showed in Figure 2. pH of the shrimp was measured using a pH meter (Mettler, Toledo, OH, USA) with a mixture of 10 g shucking shrimp muscle in 50 mL distilled water.

Total volatile basic nitrogen (TVB-N) was determined as described in [12]. Approximately 5 g shrimp was homogenized with 15 mL of 4% trichloroacetic acid (*w*/*v*). A 10 g sample was washed in the distillation flask and 1 mg magnesium oxide was added with a drop of silicone antifoam solution. Samples were boiled and distilled into 10 mL of 0.1 N HCl solutions in a 500 mL conical flask with added Tashiro indicator (Riedel-de Haen, Seelze, Germany). After distillation, the contents of the conical flask were titrated with 0.1 N NaoH. The TVB-N value was presented as milligram nitrogen per 100 g sample (mg/100 g).

Peroxide value (PV) was determined in the total lipid extracts by the AOCS method (1990) [13] and expressed as the uptake of meq active oxygen per kg lipid (meq/kg).

Thiobarbituric acid reactive substance (TBA) was measured based on the reactivity of the TBA with carbonyl compounds which may be increased in content because of lipid oxidation in shrimp. The absorbance of the extract with TBA reagent was measured at 532 nm with a UV–Vis spectrophotometer (Epoch, Biotech, VT, USA) and the constant 7.8 was used to calculate the TBA number. TBARS was expressed as mg malonaldehyde (MAD) equivalents per kg sample (mg MAD/kg).

### 2.6. Physical Quality Evaluation

The color of shrimp was analyzed using a Hunter Lab colorimeter (Hunter Associates Laboratory, Reston, VA, USA). The heads were removed from the shrimp and they were then de-shelled. Color was determined for the body muscle of the shrimp using the CIE Lab L* (lightness), a* (redness), and b* (yellowness) system (*n* = 9).

The hardness of the de-headed and de-shelled shrimp bodies was determined using a Texture Analyser (TA.XT., Stable Micro System, Godalming GU7, UK). The cell loading capacity and automated compression test mode operation of the Texture Analyser is 5 kg and 2 mm/s, respectively, with use of a stainless steel needle (P/2N) probe. Texture Exponent Software was used to process the data.

### 2.7. Microbiology Analysis

The shrimp were aseptically peeled and 10 g of peeled muscle was homogenized in 90 mL sterile saline water (0.75% NaCl) for 2 min using a Stomacher blender. The resulting homogenate was serially diluted at a ratio of 1:10 in sterile saline water. A 0.1 mL quantity of appropriate dilutions was spread on LB agar plates in triplicate and incubated at 30 °C for 48 h to determine the total viable counts (TVC) of microbial species.

### 2.8. Statistical Analyses

Data were analyzed using Minitab 17 software. ANOVA was used to compare 3 or more groups. Tukey’s test was performed at *p* < 0.05 to test for statistically significant differences. All experiments were performed in triplicate and data are expressed as means ± SD. Pearson correlation coefficients were used to analyze the presence of a relationship between parameters. The analysis was performed with the IBM SPSS Statistics 28 software package.

## 3. Results and Discussion

### 3.1. Characterization of COSP

The COSP sample contained 98.43% ash, 0.37% moisture, 0.03% protein, and 0.12% carbohydrate, respectively. The main content in the ash was calcium oxide (68%), which was the main antibacterial component in COSP [14].

### 3.2. Effect of COSP on pH in Shrimp during Refrigerated Storage

pH changes can be related to the formation of volatile amines from microbial activity and enzymatic ammonia production during refrigerated storage and as a spoilage indicator in marine products [15]. The original pH of fresh shrimp was 6.58, which increased rapidly during storage for the control group and reached 7.68 at day 12 of storage (Table 1). Mean pH increased because of the production of ammonia and trimethylamine during storage. The mean pH for the control and 0.5% COSP treatment exceeded 7.0 after 12 days of storage. Mean pH increased slowly during storage with 1.0%, 2.0%, and 4.0% treatment and reached 6.91, 6.84, and 6.80 at the end of the storage. Thus, treatment with >1.0% COSP could extend shrimp shelf-life during refrigerated storage based on the pH of the shrimp. In this study, pH values were linearly correlated with the TVC of microbial species in the corresponding samples (*R*^2^ = 0.97, 0.95, 0.96, 0.94, and 0.95 for control, 0.5%, 1.0%, 2.0%, and 4.0% COSP-treated shrimp, respectively).

### 3.3. Effect of COSP on TVB-N Content in Shrimp during Refrigerated Storage

**Figure 3:**
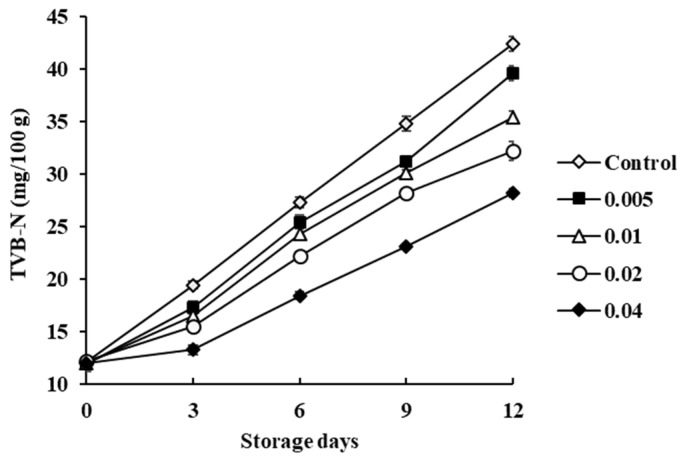
Effect of COSP on total volatile basic nitrogen (TVB-N) in white shrimp during refrigerated storage.

TVB-N content is an indicator of fishery spoilage and may be attributed to ammonia produced from bacterial catabolism of nitrogen-containing compounds, including compounds such as trimethylamine, dimethylamine, ammonia, and some other volatile basic nitrogen compounds [16]. The initial mean TVB-N content for the control was 12.14 mg/100 g and increased exponentially to 42.39 mg/100 g during storage (Figure 3). The increase is related to the activity of spoilage bacteria and endogenous enzymes. A mean TVB-N content of 35–40 mg/100 g for marine products is usually considered as spoiled [17]. Mean TVB-N content exceeded this level at 12 days refrigeration in the control, 0.5%, 1.0%, and 2.0% COSP-treated samples, whereas 4.0% COSP-treated samples were still at <30 mg/100 g. COSP treatment delayed TVB-N formation as compared with the control. Similarly, a reduction of 33–50% TVB-N content was reported for white shrimp dipped in dandelion polysaccharide and peach gum polysaccharides at the end of 12 days of storage [18]. The delay can be attributed to the protective COSP dipping solution inhibiting bacterial growth and slowing the increase in TVB-N content effectively, thus extending the preservation of shrimp.

The COSP dipping solution effectively retarded TVB-N content in shrimp. After 6 days, mean TVB-N content with 0.5% and 1.0% COSP treatment reached 25.41 mg/100 g and 24.29 mg/100 g, respectively, then markedly increased after 6 days, which was similar to the trend in TVC for microbial species, thus confirming the relationship between bacterial spoilage and TVB-N content. Mean TVB-N content was linearly correlated with TVC in control samples (*R*^2^ = 0.96) and in COSP-treated shrimp (*R*^2^ = 0.96, 0.92, 0.95, and 0.93 for 0.5%, 1.0%, 2.0%, and 4.0% COSP treatment, respectively).

### 3.4. Effect of COSP on PV in Shrimp during Refrigerated Storage

**Figure 4:**
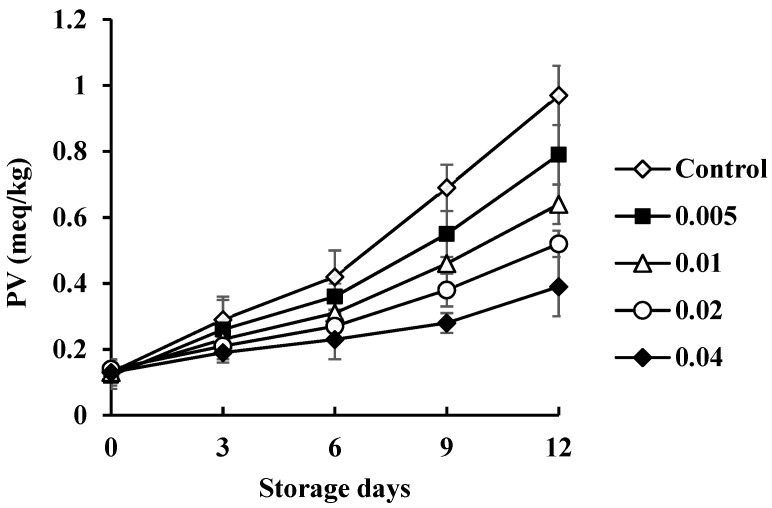
Effect of COSP on peroxide value (PV) in white shrimp during refrigerated storage.

The mean PV for shrimp with control and COSP treatments during storage is shown in Figure 4. The mean initial PV for the control was low (0.13 meq/kg) and increased within 9 days (0.69 meq/kg) of storage. *Pseudomonas* species are psychrotrophic bacteria that produce lipase and phospholipase, thus increasing free fatty acids, which are highly susceptible to oxidation and form unstable lipid hydroperoxide [17]. The increase in PV is attributed to the presence of free fatty acids in shrimp during refrigerated storage. It could favor oxidation during storage, followed by the formation of hydroperoxide or peroxide [15]. The PV scale for freshness of fish was suggested as 0–2 meq/kg for very good, 2–5 meq/kg for good, and 8–10 meq/kg for spoiled [17]. The PV increase was slower for COSP-treated than control shrimp during storage. All treated shrimp had <2 meq/kg lipids, considered an acceptable level. The mean PV in the present study indicated that COSP treatments may retard the production of initial lipid oxidation products in shrimp. These results were had the same trends with those of chitosan–gelatin coatings film, effective in retarding lipid oxidation in trout fillets [18,19]. COSP might be a good barrier to oxygen permeation and act as a resistant layer between the shrimp surface and its surrounding environment, thus decreasing the diffusion of oxygen to shrimp muscle. Mean PV was linearly correlated with the TVC of microbial species for control samples (*R*^2^ = 0.92) and COSP-treated shrimp (*R*^2^ = 0.97, 0.94, 0.93, and 0.90 for 0.5%, 1.0%, 2.0%, and 4.0% COSP treatment, respectively).

### 3.5. Effect of COSP on TBARS Content in Shrimp during Refrigerated Storage

**Figure 5:**
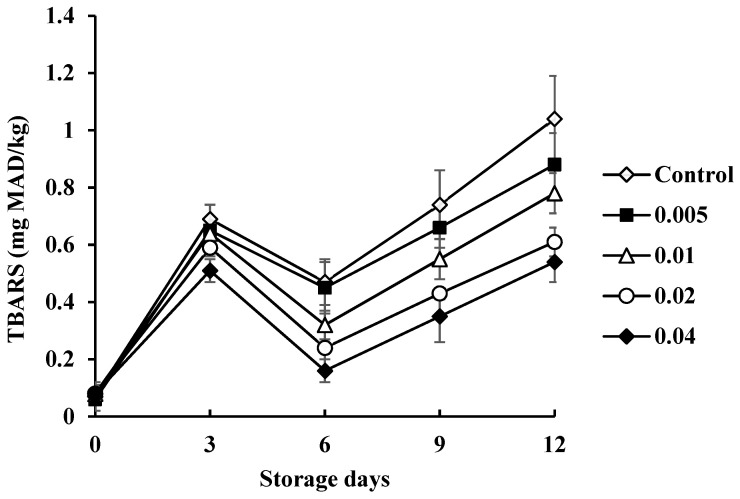
Effect of COSP on thiobarbituric acid reactive substance (TBARS) in white shrimp during refrigerated storage.

Mean TBARS content in shrimp treated with COSP during refrigerated storage is presented in Figure 5. At the beginning of storage, mean TBARS content for all samples was in the range of 0.06–0.08 mg MAD/kg meat [20]. In general, TBARS content of all samples increased up to day 3. A decrease was seen on day 6, followed by a gradual increase up to 12 days of storage. The decreased TBARS content on day 6 was more likely due to loss of secondary lipid oxidation products previously formed within the first 3 days. Lipid oxidation is one of the deteriorative reactions causing the unacceptability of fish and shrimp products which can be initiated by autoxidation and enzymatic reactions involving lipoxygenase, peroxidase, and microbial enzymes [21]. The increase in TBARS content was lower with COSP treatment as compared with the control. A TBARS content of 1–2 mg MAD/kg in the fishery is related to an unpleasant odor. The content reached the limit only for the control at the end of storage. TBARS content indicates the concentration of final lipid oxidation products. Lowered lipid oxidation was in accordance with lower microbial growth in shrimp treated with COSP for some doses (Table 2). COSP might have an antioxidative effect and show oxygen barrier properties in shrimp muscle during extended refrigerated storage; these could also be found during the preservation of Gourami fish fillets and chicken meat [22,23]. The antioxidant mechanism of COSP could be interpreted as follows: the primary amino acids of shrimp form a stable state with malondialdehyde derived from the breakdown of lipids during oxidation. We found a linear correlation between the TVC of microbial species and mean TBARS in control samples (*R*^2^ = 0.98) and COSP-treated shrimp (*R*^2^ = 0.92, 0.91, 0.94, and 0.93 for 0.5%, 1.0%, 2.0%, and 4.0% COSP treatment, respectively).

### 3.6. Effect of COSP on the Physical Quality of Shrimp during Refrigerated Storage

**Figure 6:**
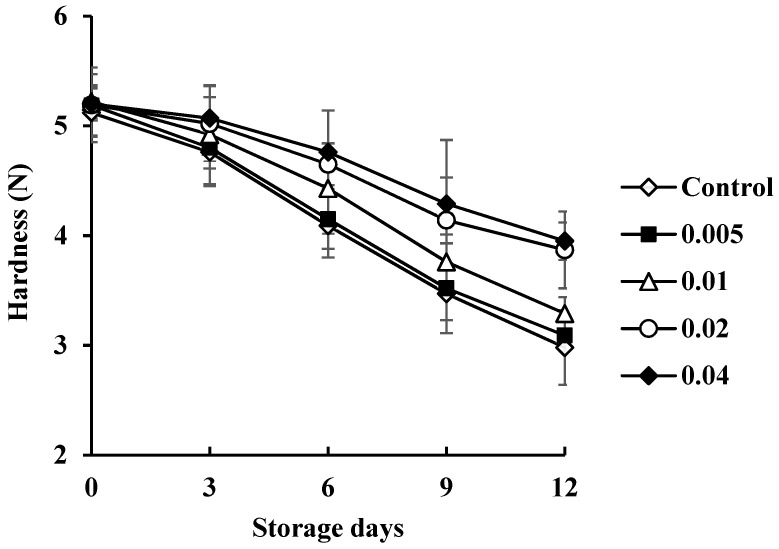
Changes in hardness of white shrimp treated with COSP during refrigerated storage.

Hardness is one of the important textural parameters for consumer acceptability. The hardness changes of the control and COSP-treated shrimp during refrigerated storage are shown in Figure 6. The differences in hardness can result from increased bacterial content of aerobic bacterial, and interactions between COSP dipping solution and shrimp tissue caused changes in hardness. COSP-treated shrimp tended to be harder than the control, which indicates that COSP treatment was efficient in improving the physical properties of shrimp. Similar results were found for chitosan-coated oil sardine, chitosan–gelatin-coated shrimp, and green tea extract-treated shrimp [24,25,26,27]. Hardness was correlated linearly with the TVC of microbial species for control shrimp (*R*^2^ = −0.97) and COSP-treated shrimp (*R*^2^ = −0.94, −0.92, −0.96, and −0.97 for 0.5%, 1.0%, 2.0%, and 4.0% COSP treatment, respectively) and control shrimp.

**Figure 7:**
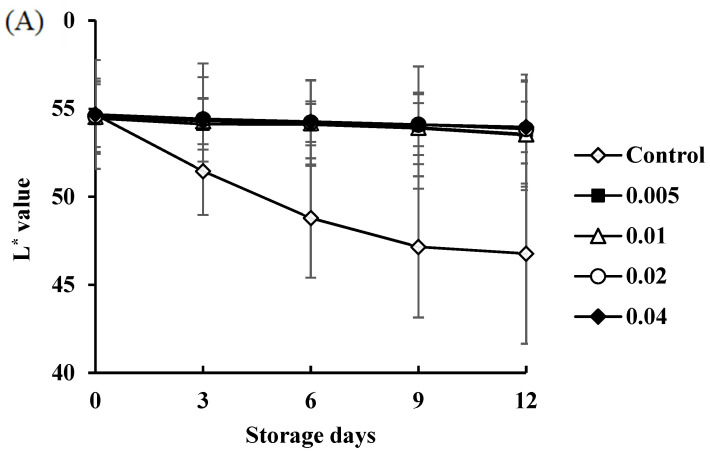

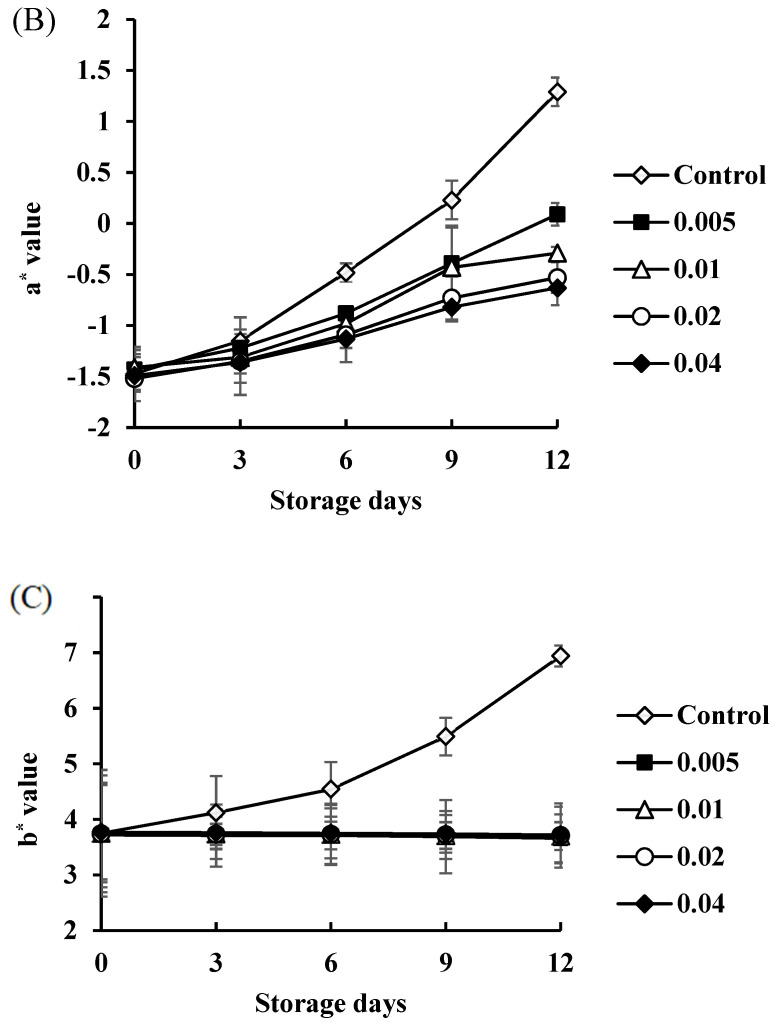
Changes in color properties of white shrimp treated with COSP during refrigerated storage. (**A**) L* values; (**B**) a* values; (**C**) b* values.

The color analysis of COSP-treated and control shrimp during refrigerated storage is shown in Figure 7A. The L* value of all samples decreased significantly during storage, as seen by the appearance of black spots. The decrease in L* value with time was slower for COSP-treated shrimp than the control. At the end of storage, the values were about eight points below the initial value for the control, but only two points below for COSP-treated shrimp. In previous studies, the presence of chitosan, gelatin, polysaccharides, and plant extract in dipping solution prevented the decreased lightness of color of shrimp [16]. The L* values correlated with the TVC of microbial species in the control shrimp (*R*^2^ = −0.95) and COSP-treated shrimp (*R*^2^ = −0.93, −0.91, −0.94, and −0.92 for 0.5%, 1.0%, 2.0%, and 4.0% COSP treatment, respectively).

**Figure 8:**
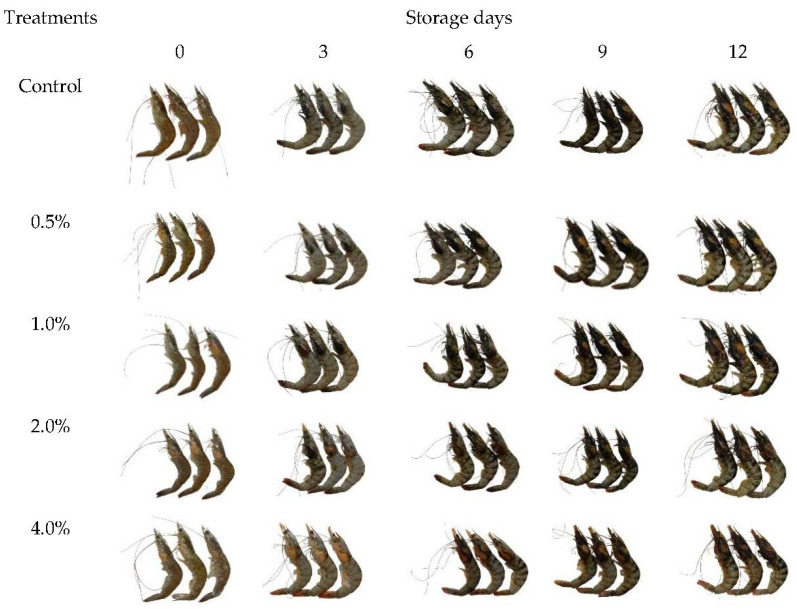
The appearance of white shrimp treated with COSP during refrigerated storage.

The a* values increased with increasing storage time, with a slower increase for COSP-treated shrimp, as shown in Figure 7B. The same increasing tendency with time for the a* value was observed in shrimp; the color alters with the oxidation of lipids due to hydrolysis of astaxanthin by endogenous enzymes which is then released into the protein matrixes in muscle tissues [24]. The a* value changes might be due to the enhanced passage of oxygen through the COSP barrier because of intermolecular interactions in the generated structural matrix. COSP treatment did not result in any significant changes in b* values, as shown in Figure 7C. COSP treatment could have contributed to the maintenance of yellowness throughout the storage period (Figure 8). Avocado seed extracts prevented an increase in the redness and yellowness of shrimp muscle during storage [28]. Use of a COSP coating had no important effect on yellowness but did increase lightness and redness for coated cod patties [29]. The a* value was correlated with the TVC of microbial species (*R*^2^ = 0.98, 0.94, 0.91, 0.95, and 0.97) in the control and 0.5%, 1.0%, 2.0%, and 4.0% COSP-treated shrimp, respectively. The b* value was correlated with the TVC of microbial species (*R*^2^ = 0.96) in control shrimp.

### 3.7. Microbiological Analysis of Refrigerated Shrimp 

The TVCs of microbial species in shrimp with different COSP treatments during storage at 4 °C are presented in Table 2. The mean initial number of bacteria in fresh shrimp ranged from 4.57 × 10^1^ CFU/g to 1.47 × 10^2^ CFU/g, close to the results of 10^2^ to 10^3^ CFU/g reported by Mace (2014) [30] and Liu (2016) [31]. During storage at 4 °C, the mean TVC of the control shrimp rapidly increased to 2.34 × 10^4^ CFU/g after 6 days of storage, faster than COSP-treated shrimp. From day 6 to day 12, the mean TVC in control shrimp increased quickly, to 1.58 × 10^8^ CFU/g on day 12. COSP-treated shrimp showed significantly inhibited growth of bacteria during storage; the mean TVC with COSP 0.5% treatment was 8.22 × 10^7^ CFU/g on day 12, more than 10 times lower than that of the control, and mean TVC with COSP at 1.0%, 2.0%, and 4.0% was 5.21 × 10^6^ CFU/g, 3.16 × 10^5^ CFU/g, and 6.22 × 10^4^, CFU/g, respectively, also lower than the control value, and still under the organoleptically detectable level of 1 × 10^7^ CFU/g [17].

Oyster shells consist of about 95% calcium carbonate (CaCO_3_). After calcining, CaCO_3_ is converted into calcium oxide (CaO) [4]. This has a good antibacterial effect via increased pH value during food preservation. Therefore, COSP is often used as a natural antibacterial agent. COSP could inhibit the growth of *Staphylococcus aureus*, *Escherichia coli*, *Listeria*, *Salmonella*, *Cactus bacillus*, *Micrococcus luteus*, *Aspergillus niger*, and *Penicillium funiculosum* [7]. It can be applied to preserve fresh-cut fruits and vegetables, in whole fish treatment, toothbrushes, antibacterial air conditioning filters, antibacterial fabrics, and cleaning cloths. High-temperature treatment of oyster shell powder (calcined at 900 °C for 5 h) at 1.25% had antibacterial effects on food pathogens (*E. coli*, *S. aureus*, *Salmonella typhimurium*, and *Vibrio parahaemolyticus*), and the bacteriostatic zone can reach >10.0 mm in diameter. Using oyster shell powder burned at 800 °C for 5 h, a concentration of 1% could reduce the weight of mold (*A. niger*) hyphae. Hard bean curd and frozen tofu treated with 0.5% shell powder (calcined at 900 °C for 5 h) had a lower total bacterial count than commercially available hard bean curd after 28 days, reduced to 4.19 × 10^6^ and 4.16 × 10^5^ CFU/mL, respectively [14].

## 4. Conclusions

This study investigated the effect of COSP treatment on the quality of refrigerator-stored white shrimp (*L. vannamei*). This is the first report on the biochemical and physical attributes of COSP-treated refrigerator-stored shrimp. COSP treatment affected the pH, TVB-N, PV, TBARS, hardness, color (L*, a* and b* values), and TVC of microbial species in refrigerator-stored shrimp. The mechanism of preservation for COSP treatment might be the CaOH that was formed from CaO in the dipping solution, which resulted in increased pH and inhibited the growth of bacteria, as indicated by a lower increase in TVC in the COSP-treated shrimp than in the controls, and changed the activity of enzymes in shrimp muscle, thus resulting in a lower increase in biochemical and physical attributes. Hence, COSP dipping solution treatment was effective in reducing the spoilage and prolonging the shelf-life of seafood, which might provide processors or distributors with viable options for preservatives to improve the microbiological safety and quality of shrimp.

## Figures and Tables

**Table 1:** Effect of calcined oyster shell powder (COSP) on pH in white shrimp during refrigerated storage.

Attributes	COSP (%)	Storage Days				
		0	3	6	9	12
pH	Control	6.58 ± 0.06 ^a^	6.67 ± 0.03 ^a^	7.01 ± 0.06 ^a^	7.48 ± 0.07 ^a^	7.68 ± 0.09 ^a^
	0.5%	6.62 ± 0.11 ^a^	6.64 ± 0.02 ^a^	6.90 ± 0.07 ^b^	7.37 ± 0.05 ^a^	7.61 ± 0.02 ^a^
	1.0%	6.56 ± 0.09 ^a^	6.60 ± 0.04 ^a^	6.83 ± 0.03 ^b^	6.88 ± 0.04 ^b^	6.91 ± 0.04 ^b^
	2.0%	6.61 ± 0.04 ^a^	6.62 ± 0.06 ^a^	6.79 ± 0.09 ^bc^	6.82 ± 0.05 ^b^	6.84 ± 0.06 ^b^
	4.0%	6.67 ± 0.13 ^a^	6.63 ± 0.03 ^a^	6.72 ± 0.05 ^c^	6.78 ± 0.09 ^b^	6.80 ± 0.05 ^b^

Data are means ± SD from triplicate experiments. ^a–c^ Different letters indicate significant (*p* < 0.05) differences in the same row.

**Table 2:** Effect of COSP on total viable count (TVC) of microbial species in white shrimp during refrigerated storage.

	COSP (%)	Storage Days				
		0	3	6	9	12
***TVC***	Control	1.47 ± 0.06 ^a^ × 10^2^	1.13 ± 0.22 ^a^ × 10^3^	2.34 ± 0.21 ^a^ × 10^4^	4.66 ± 0.22 ^a^ × 10^6^	1.58 ± 0.32 ^a^ × 10^8^
	0.5%	1.21 ± 0.11 ^b^ × 10^2^	6.34 ± 0.23 ^b^ × 10^2^	9.17 ± 1.50 ^b^ × 10^3^	2.82 ± 0.14 ^b^ × 10^5^	8.22 ± 0.66 ^b^ × 10^7^
	1.0%	1.07 ± 0.15 ^b^ × 10^2^	3.77 ± 0.12 ^c^ × 10^2^	4.67 ± 0.35 ^c^ × 10^3^	6.15 ± 0.18 ^c^ × 10^4^	5.21 ± 0.23 ^c^ × 10^6^
	2.0%	7.23 ± 0.56 ^c^ × 10^1^	3.14 ± 0.06 ^d^ × 10^2^	1.03 ± 0.15 ^d^ × 10^3^	3.25 ± 0.18 ^d^ × 10^4^	3.16 ± 0.54 ^d^ × 10^5^
	4.0%	4.57 ± 0.23 ^d^ × 10^1^	6.25 ± 0.12 ^e^ × 10^1^	3.67 ± 0.22 ^e^ × 10^2^	5.25 ± 0.18 ^e^ × 10^3^	6.22 ± 0.15 ^e^ × 10^4^

Data are means ± SD from triplicate experiments. ^a–e^ Different letters indicate significant (*p* < 0.05) differences in the same row.

## Data Availability

MDPI Research Data Policies at https://www.mdpi.com/ethics (15 January 2022).
